# The multikinase inhibitor RXDX-105 is effective against neuroblastoma *in vitro* and *in vivo*

**DOI:** 10.18632/oncotarget.27259

**Published:** 2019-10-29

**Authors:** Sean M. Flynn, Jacqueline Lesperance, Andrew Macias, Nikki Phanhthilath, Megan Rose Paul, Jong Wook Kim, Pablo Tamayo, Peter E. Zage

**Affiliations:** ^1^ Department of Surgery, University of California San Diego, La Jolla, CA, USA; ^2^ Department of Pediatrics, Division of Hematology-Oncology, University of California San Diego, La Jolla, CA, USA; ^3^ Peckham Center for Cancer and Blood Disorders, Rady Children's Hospital, San Diego, CA, USA; ^4^ Department of Medicine, Moores Cancer Center, University of California San Diego, La Jolla, CA, USA

**Keywords:** neuroblastoma, RXDX-105, CEP-32496, RET, BRAF

## Abstract

Neuroblastoma is the most common extracranial solid tumor of childhood and accounts for 15% of all pediatric cancer-related deaths. New therapies are needed to improve outcomes for children with high-risk and relapsed tumors. Inhibitors of the RET kinase and the RAS-MAPK pathway have previously been shown to be effective against neuroblastoma, suggesting that combined inhibition may have increased efficacy. RXDX-105 is a small molecule inhibitor of multiple kinases, including the RET and BRAF kinases. We found that treatment of neuroblastoma cells with RXDX-105 resulted in a significant decrease in cell viability and proliferation *in vitro* and in tumor growth and tumor vascularity *in vivo*. Treatment with RXDX-105 inhibited RET phosphorylation and phosphorylation of the MEK and ERK kinases in neuroblastoma cells and xenograft tumors, and RXDX-105 treatment induced both apoptosis and cell cycle arrest. RXDX-105 also showed enhanced efficacy in combination with 13-*cis*-retinoic acid, which is currently a component of maintenance therapy for children with high-risk neuroblastoma. Our results demonstrate that RXDX-105 shows promise as a novel therapeutic agent for children with high-risk and relapsed neuroblastoma.

## INTRODUCTION

Neuroblastoma is the most common extracranial solid tumor of childhood and accounts for 15% of all pediatric cancer-related deaths [1]. Current treatment for children with high-risk neuroblastoma includes intensive multimodal therapy with significant associated morbidity [2, 3]. Despite initial responses to therapy, children with high-risk neuroblastoma tumors suffer from frequent relapses, and relapsed tumors are often refractory to current treatment. Thus, overall outcomes are poor for this patient population, with overall survival rates less than 20% [4–6]. New therapeutic agents directed against novel targets and mechanisms are needed to address the clinical needs of these patients.

The REarranged during Transfection (*RET*) gene encodes a receptor tyrosine kinase that is a known oncoprotein, with oncogenic mutations identified in multiple tumor types. RET is expressed on neural crest-derived cells and is important for the maturation of sympathetic neurons [7]. RET has also been shown to be expressed on neuroblastoma tumor cells [8], and transgenic mice overexpressing RET develop neuroblastoma tumors [9]. RET expression is also associated with increased neuroblastoma metastases *in vivo* [10], and RET expression is higher in neuroblastoma tumors from patients with stage 4 and high-risk disease [11]. These results suggest that RET may play an important role in neuroblastoma cell survival, proliferation, and metastasis, and therefore RET is an appealing target for novel therapeutic agents.

The RAS-RAF-MAPK pathway is activated downstream of RET and other receptor tyrosine kinases and is likewise frequently mutated in numerous types of human cancer [12]. Single mutations in the RAS-MAPK pathway are uncommon in neuroblastoma tumors at the time of initial diagnosis, with mutations of BRAF seen in approximately 1% of tumors and other RAS-MAPK pathway mutations only found in approximately 3–5% [13–14]. Recent investigations, however, identified a majority of relapsed neuroblastoma tumors with mutations suspected to activate the RAS-MAPK pathway [15, 16]. These results suggest that the RAS-MAPK pathway potentially plays a role in the resistance of neuroblastoma tumors to upfront therapy and that RAS-MAPK pathway inhibition may be most effective in children with relapsed neuroblastoma.

**Figure 1:**
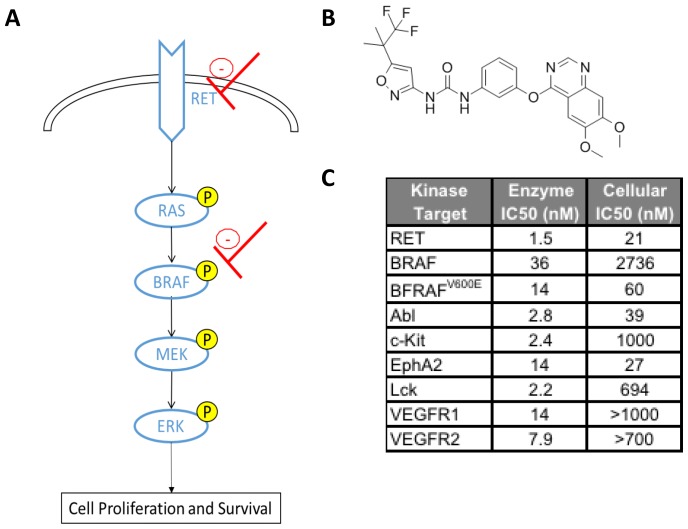
(**A**) The RET, RAS-RAF-MAPK pathway with sites of RXDX-105 inhibition in red. (**B**) RXDX-105 (CEP-32496) structure. (**C**) RXDX-105 inhibition of potential target kinases (adapted from [17]).

RXDX-105 is a novel, small molecule, multi-kinase inhibitor with potent activity against wild type RET, RET fusions, and RET activating mutations as well as other kinases [17] (Figure 1). RXDX-105 is orally available and has been tolerated well by adults in phase I/Ib clinical trials [18, 19]. Given the evidence for the roles of RET and the RAS-MAPK pathway in neuroblastoma pathogenesis and treatment resistance, we hypothesize that RXDX-105 should have significant antitumor effects in *in vitro* and *in vivo* models of neuroblastoma and may be a promising new therapy for children with relapsed neuroblastoma.

## RESULTS

### RXDX-105 decreases neuroblastoma cell viability and proliferation

**Figure 2:**
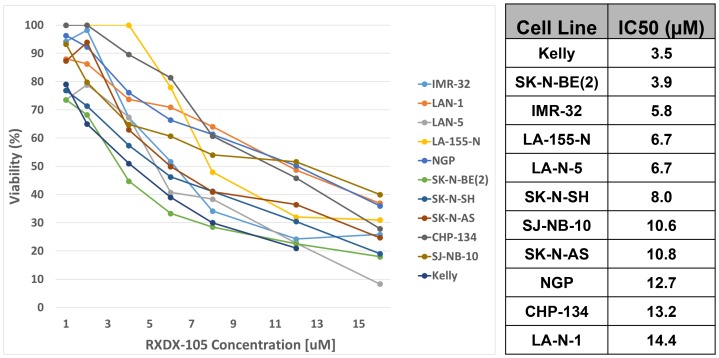
RXDX-105 decreases neuroblastoma cell viability and proliferation. Cell viability was assessed with alamarBlue^TM^ assays performed after 72 hours of incubation with RXDX-105, and dose-response curves (left) and calculated IC50 values (right) are shown.

To determine the effects of RXDX-105 on neuroblastoma cell viability, a set of 11 established neuroblastoma cell lines, representing a range of biological and cytogenetic phenotypes (Supplementary Table 1), were cultured in physiologically relevant concentrations of RXDX-105 [19]. Cell viability was assessed with alamarBlue^TM^ assays performed after 72 hours of incubation with the drug. IC50 values were calculated and ranged from 3.5 μM and 14.4 μM (Figure 2), suggesting that neuroblastoma cells are sensitive to RXDX-105 at physiologically achievable doses. We also assessed the effects of RXDX-105 on cell confluence utilizing continuous live cell imaging. Cell confluence in treated cells compared to untreated cells was calculated at 72 hours. IC50 values for confluence were similar to those calculated from cell viability assays (Supplementary Table 2). No apparent associations were observed between known cytogenetic and biologic features of the neuroblastoma cell lines, including *MYCN* amplification or other cytogenetic abnormalities or p53 mutations, and sensitivity to RXDX-105.

### RXDX-105 induces neuroblastoma cell apoptosis and cell cycle arrest

**Figure 3:**
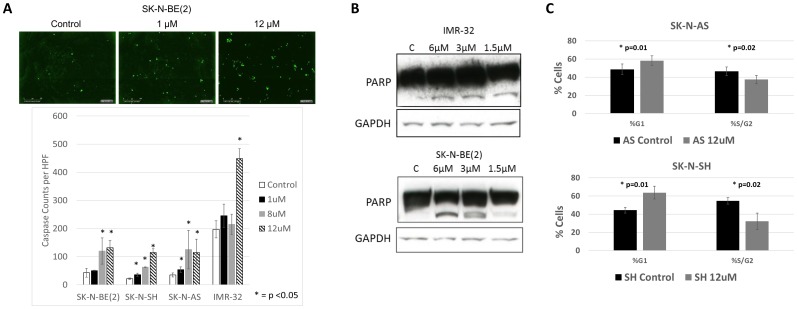
RXDX-105 induces neuroblastoma cell apoptosis and cell cycle arrest. (**A**) Neuroblastoma cells were plated and treated with vehicle control or decreasing doses of RXDX-105 with additional caspase 3/7 reagent. Cells were monitored with continuous live cell imaging and total caspase cleavage was determined by counting sites of activated caspases (in green) at 72 hours. (**B**) Cell lysates were assessed for PARP cleavage by Western blot after 24 hours of RXDX-105 treatment. (**C**) The effect of RXDX-105 treatment on cell cycle was assessed using flow cytometry for DNA content after 24 hours of treatment.

To assess the mechanisms through which RXDX-105 inhibited cell viability and reduced confluence, we performed assays to measure apoptosis in neuroblastoma cells treated with RXDX-105 and equivalent controls. RXDX-105 treatment resulted in significantly increased caspase and PARP cleavage in all cell lines tested in a dose dependent manner (Figure 3), suggesting that RXDX-105 exposure induces apoptosis in neuroblastoma cells.

To determine the effects of RXDX-105 on cell cycle progression, neuroblastoma cells were treated with RXDX-105 and analyzed by flow cytometry for DNA content. 24 hours of RXDX-105 exposure resulted in a significant increase in the percentage of cells in the G0/G1 phase and a concurrent decrease in the percentage of S/G2 phase cells (Figure 3), suggesting that both induction of apoptosis as well as cell cycle arrest underlie the decreased viability and confluence seen in RXDX-105-treated neuroblastoma cells.

### RXDX-105 inhibits target kinases and intracellular signaling pathways

**Figure 4:**
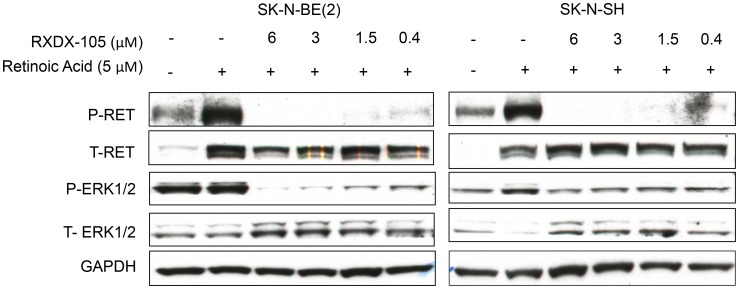
RXDX-105 inhibits target kinases and intracellular signaling pathways. Neuroblastoma cell lines were treated with either vehicle or decreasing doses of RXDX-105 for 24 hours in combination with 5 μM 13-*cis*-retinoic acid to stimulate RET phosphorylation. Cells were lysed and Western blots were performed to assess RET and ERK expression and phosphorylation. GAPDH expression is shown as a loading control.

RXDX-105 is a potent inhibitor of multiple kinases, including the RET and BRAF kinases (Figure 1) [17]. To investigate whether RXDX-105 is able to inhibit the RET kinase and the RAS-MAPK pathway in neuroblastoma cells, we evaluated a panel of neuroblastoma cell lines for RET, MEK, and ERK expression and phosphorylation after 24 hours of treatment with RXDX-105. RET phosphorylation was inhibited with RXDX-105 treatment in a dose dependent manner, while levels of total RET remained relatively stable. RET phosphorylation was also increased after the addition of 5 μM 13-*cis*-retinoic acid, which was also inhibited by RXDX-105 (Figure 4). RXDX-105 treatment also resulted in significantly decreased MEK and ERK phosphorylation, while levels of total ERK remained the same or slightly increased (Figure 4, Supplementary Figure 1).

### Sensitivity to RXDX-105 is related to RAS-MAPK pathway gene expression

Cell lines with gene expression data available were analyzed to assess for differences in the expression of genes for intracellular signaling pathway proteins that might explain the differences seen in sensitivity to RXDX-105. The median IC50 for all cells tested was 8.0 μM. Cells with IC50s less than 8.0 μM, including Kelly, SK-N-AS, and SK-N-BE (2), trended toward having increased expression of RAS-MAPK pathway genes (Supplementary Figure 2), while there was no appreciable trend between RET gene expression and RXDX-105 sensitivity (data not shown).

### RXDX-105 demonstrates enhanced efficacy when combined with 13-cis-retinoic acid

**Figure 5:**
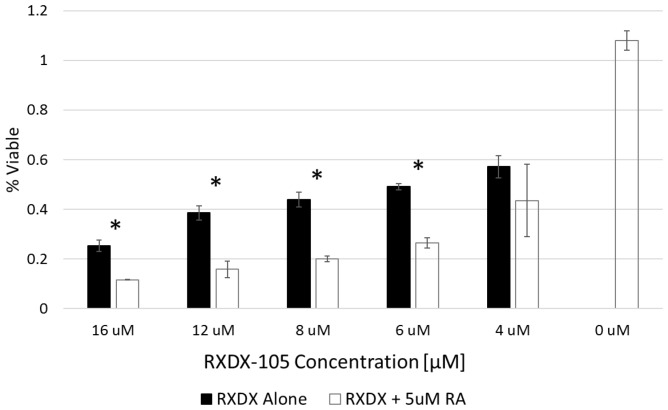
RXDX-105 efficacy is enhanced when combined with 13-*cis*-retinoic acid. SK-N-BE(2) neuroblastoma cells were treated with decreasing doses of RXDX-105 alone or in combination with 5 μM 13-*cis*-retinoic acid for 72 hours and cell viability was assessed with an alamarBlue™ assay. Cell viability at each dose of RXDX-105 is shown.

We have previously demonstrated synergistic efficacy of the combinations of RET inhibitors with 13-*cis*-retinoic acid, a vitamin A analog currently used as maintenance therapy in children with high-risk neuroblastoma [20, 21]. To determine the efficacy of the combination of RXDX-105 and 13-*cis*-retinoic acid, we plated cells with and without 5 μM 13-*cis*-retinoic acid with increasing doses of RXDX-105 for 72 hours before performing alamarBlue^TM^ assays. 13-*cis*-retinoic acid had no significant effect on cell viability as a single agent, but the combination of RXDX-105 with 13-*cis*-retinoic acid resulted in a significant decrease in cell viability in all cells tested, compared to RXDX-105 alone (Figure 5; Supplementary Figure 3).

### RXDX-105 inhibits neuroblastoma xenograft tumor growth

**Figure 6:**
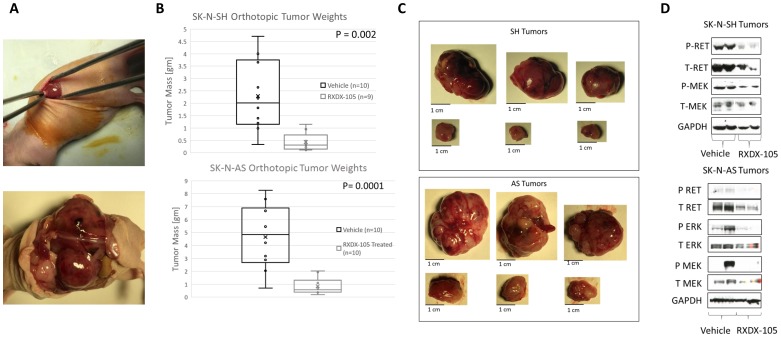
RXDX-105 inhibits tumor xenograft growth and inhibits RET phosphorylation and intracellular signaling in orthotopic neuroblastoma tumors. (**A**) SK-N-AS and SK-N-SH cells were injected into the left adrenal glands of nude mice as shown. Mice were then randomized and treated once daily orally with vehicle control or RXDX-105. SK-N-AS tumors were harvested at 4 weeks and SK-N-SH tumors at 6 weeks, based on tumor growth rates. (**B**) Final tumor weight was measured in harvested tumors and weights of tumors from treated mice were compared to weights of tumors from control mice. (**C**) Images and measurements from harvested tumors are shown, with untreated tumors in the top rows and treated tumors in the bottom rows. (**D**) Tumor cell lysates were analyzed by Western blot for expression and phosphorylation of RET, MEK and ERK in tumors from treated and control mice.

To evaluate the efficacy of RXDX-105 against neuroblastoma tumors *in vivo*, we utilized an orthotopic xenograft model, injecting neuroblastoma cells into the exposed left adrenal glands of immunocompromised mice (Figure 6) [21]. RXDX-105 was well tolerated without any decrease in mouse weight or other appreciable side effects (Supplementary Figure 4). Treatment with RXDX-105 resulted in significant reduction in final tumor weights in both SK-N-AS (4.7 g ± 2.5 g vs. 0.85 g ± 0.65 g, *p* = 0.0001) and SK-N-SH (2.3g ± 1.4 g vs. 0.43 g ± 0.37 g, *p* = 0.002) xenograft tumors after 4 and 6 weeks of treatment respectively (Figure 6, Supplementary Figure 5).

### RXDX-105 inhibits RET phosphorylation and intracellular signaling downstream of BRAF in orthotopic neuroblastoma tumors

To determine whether RXDX-105 treatment was also able to inhibit RET phosphorylation as well as the RAS-MAPK pathway in mice with neuroblastoma xenograft tumors, we evaluated lysates of harvested tumors by Western blots for total and phosphorylated RET, MEK, and ERK. Neuroblastoma tumors from mice treated with RXDX-105 demonstrated decreased levels of phosphorylated RET as well as decreased phosphorylation of MEK and ERK (Figure 6), suggesting that the mechanism of efficacy of RXDX-105 *in vivo* is likely similar to its mechanism *in vitro*.

### RXDX-105 treatment leads to decreased angiogenesis *in vivo*

**Figure 7:**
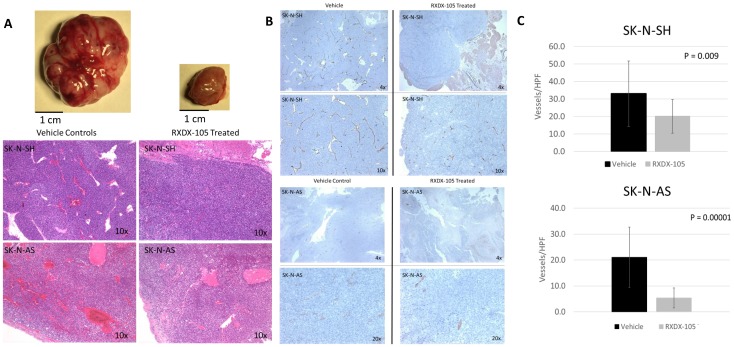
RXDX-105 treatment leads to decreased angiogenesis *in vivo*. (**A**) Drug treated tumor samples were noted to be less vascular and hemorrhagic on gross appearance and H&E staining. (**B**) Tumor samples were immunostained for vascular endothelial cells with anti-CD31 antibodies and photographed at 10× magnification. (**C**) Mean vessel density was calculated using the number of observed vessels per high power field and compared in both treated and untreated tumors.

We noted a significant difference in the gross appearance of RXDX-105-treated and vehicle control-treated tumors in orthotopic neuroblastoma tumor models, with the RXDX-105-treated tumors being notably paler in appearance and less friable and hemorrhagic on gross appearance (Figure 6). To evaluate the effects of RXDX-105 on neuroblastoma tumor vascularity, tumor samples were immunostained for vascular endothelial cells with anti-CD31 antibodies and mean vessel density was calculated in treated and untreated tumors. Mean vessel density was significantly decreased in tumors from mice treated with RXDX-105 compared to control tumors, suggesting the efficacy of RXDX-105 against neuroblastoma tumors is mediated in part by a potent anti-angiogenic effect (Figure 7).

## DISCUSSION

New therapies are needed for children with high-risk and relapsed neuroblastoma who continue to have poor outcomes despite intensive treatment. We have demonstrated that the novel kinase inhibitor RXDX-105 is effective against neuroblastoma in both *in vitro* and *in vivo* models.

RET has been previously shown to be highly expressed in neuroblastoma cells, and constitutively activated RET enhances neuroblastoma metastases *in* vivo [8, 10]. A recently described subset of approximately 10% of primary neuroblastoma tumors with ALK mutations has been shown to be driven through RET upregulation [22]. Our results add further evidence that RET plays a significant role in neuroblastoma cell proliferation and survival and that the RET kinase represents a promising therapeutic target. Prior studies of RET inhibitors have also demonstrated efficacy against neuroblastoma *in vitro* and *in vivo* through decreasing neuroblastoma viability and induction of apoptosis [11, 20, 21]. However, these inhibitors, like RXDX-105, also inhibit other important targets in critical signaling pathways which likely contribute significantly to their antitumor effects. The effects of RXDX-105 in neuroblastoma are potentially due to the combination of the effects of inhibition of multiple kinases, including RET, BRAF, Abl, and EphA2 (Figure 1), and further studies are needed to clarify the relative roles of these disparate kinases in the responses of neuroblastoma cells to RXDX-105.

Prior studies have also demonstrated the efficacy of MEK inhibitors against neuroblastoma models [15, 23, 24]. Our results demonstrate that RXDX-105 inhibits both RET and the RAS-MAPK pathway, which likely both contribute to the observed potent *in vitro* and *in vivo* antitumor effects and suggest that RET and its downstream RAS-MAPK pathway are involved in neuroblastoma pathogenesis. Our results are further supported by the trend seen towards increased cell line sensitivity to RXDX-105 in cell lines with increased RAS-MAPK pathway gene expression, and there was no observed relationship between *MYCN* amplification or other cytogenetic abnormalities or p53 mutations with responses to RXDX-105.

Our results have also shown that RXDX-105 treatment of neuroblastoma tumors results in a decrease in tumor vascularity. Prior studies have shown that treatment of neuroblastoma tumors with vandetanib, a RET, VEGFR, and EGFR inhibitor, resulted in a significant decrease in tumor angiogenesis *in* vivo [20]. RXDX-105 has previously been shown to have minimal inhibitory effect on VEGFR, potentially minimizing some of the known dose-limiting side effects of previously studied VEGFR inhibitors, such as hypertension [17]. It has previously been suggested that RET stimulation may promote angiogenesis through the activation of pro-inflammatory mediators and the recruitment of primary immune cells to the tumor microenvironment, which subsequently promotes angiogenesis and suggests that RET inhibition inhibits angiogenesis by blocking this activation [26]. Our results demonstrating an antiangiogenic effect of RXDX-105 in immunocompromised mouse models, however, suggest that RET, and possibly the RAS-MAPK pathway, may play previously undescribed roles in neuroblastoma tumor angiogenesis, although direct antiangiogenic effects of EphA2 inhibition, in addition to inhibition of VEGFR1 and VEGFR2, also cannot be completely ruled out at the concentrations likely achieved in our mice treated with RXDX-105.

Our results also demonstrate that the combination of RXDX-105 and 13-*cis*-retinoic acid is more effective than either agent alone. 13-*cis*-retinoic acid is a Vitamin A analog currently used for maintenance therapy in treatment regimens for children with high-risk neuroblastoma [3], and 13-*cis*-retinoic acid has been shown to induce neuroblastoma tumor differentiation, decrease *MYCN* expression, and decrease neuroblastoma cell proliferation [27–30]. 13-*cis*-retinoic acid treatment has been shown to induce dependence on neurotrophin and glial-derived neurotrophic factor signaling through the RET kinase [31, 32], suggesting that the observed synergistic effects of RET inhibition with 13-*cis*-retinoic acid are possibly due to the induction of oncogenic addiction to RET signaling [33, 34], which subsequently sensitizes neuroblastoma cells to RET inhibition.

In summary, we have demonstrated that the novel multikinase inhibitor RXDX-105 is effective against neuroblastoma tumors both *in vitro* and *in vivo*. RXDX-105 treatment of neuroblastoma cells and xenograft tumors results in inhibition of RET and the RAS-MAPK pathway, adding further evidence to the critical roles these proteins and pathways play in neuroblastoma tumorigenesis. Given this demonstrated efficacy, further preclinical and clinical testing of RXDX-105 in patients with relapsed neuroblastoma is warranted.

## MATERIALS AND METHODS

### Cells and culture conditions

The neuroblastoma cell lines SK-N-AS, SK-N-SH, IMR-32, SK-N-BE (2), LA-N-1, LA-N-5, LA1-55N, Kelly, NGP, CHP-134, and SJ-NB-10 have been previously described [20, 21, 24]. Cell lines were maintained in RPMI-1640 (Mediatech Inc, Manassas, VA, USA) with 10% fetal bovine serum (Omega, Tarzana, CA, USA), L-glutamine, non-essential amino acids (Mediatech Inc), sodium pyruvate (Mediatech Inc), and Hyclone antibiotic/antimycotic (Fisher Scientific, Hampton, NH, USA) at 37°C in 5% CO_2_.

### RXDX-105

RXDX-105 was generously provided by Ignyta, Inc. (San Diego, CA, USA). *In vitro* experiments were performed with RXDX-105 (free base), which was dissolved in dimethylsulfoxide (DMSO) to create a 25 mM stock solution, which was maintained at 4° C for 1 week at a time and protected from light. *In vivo* experiments were performed with RXDX-105 (HCl salt), which was dissolved in 22% (2-Hydroxypropyl)-β-cyclodextrin (HPBCD; Sigma Aldrich, St. Louis, MO, USA) stored at 4°C for 1 week at a time.

### Cell confluence assay

Cells were plated in 96-well plates at seeding densities between 5,000–10,000 cells/well for 24 hours. Cells were then treated with varying concentrations of RXDX-105 or equivalent doses of DMSO for control. Plates were placed in the Incucyte Zoom^TM^ continuous live cell imaging system (Essen Bioscience, Ann Arbor, MI, USA) and phase contrast images were taken every 6 hours at 10× magnification for up to 96 hours. Cell growth curves were generated from percent cell confluence acquired from the Incucyte Zoom^TM^ analyzer. Replicates of at least three wells were used for each drug concentration for every experiment and each experiment was repeated at least three independent times. IC50 values were derived using best-fit trend lines and the values were calculated using the appropriate curve-fit equations.

### CASPASE 3/7 apoptosis assay

Cells were plated in 96-well plates at seeding densities of 5,000 cells/well and treated with RXDX-105 at different concentrations as above. Caspase 3/7 Reagent (Essen Bioscience) was added 24 hours after plating. Plates were placed in the Incucyte Zoom™ live cell imaging system and phase contrast images as well as green fluorescent images were taken every 6 hours at 10× magnification for 72 hours. Fold apoptosis was generated from green signal counts measured by the Incucyte Zoom™ analyzer for treated cells and normalized to the green signal count measured for control wells. Replicates of at least three wells were used for each drug concentration for every experiment.

### Cell viability assay

Cells were plated in 96-well plates at a seeding density of 5,000 cells/well and incubated overnight. Cells were then treated with varying concentrations of RXDX-105 or equivalent doses of DMSO for control. After 72 hours of incubation with RXDX-105, alamarBlue™ Cell Viability Reagent (Invitrogen, Carlsbad, CA, USA) was added at 10% of total volume. Cells were then incubated from 1–6 hours at 37° C protected from light. Plates were read on Tecan i200 spectrophotometer at 590 nm. Viability as determined by signal intensity was normalized to control wells.

### Combinations with 13-*cis*-retinoic acid

SK-N-BE (2) neuroblastoma cells were plated in 96 well plates at approximately 50% confluence, given 24 hours to adhere, and then were treated with either escalating concentrations of RXDX-105 alone or in combination with 5 μM 13-*cis*-retinoic acid. SK-N-BE (2), SK-N-SH, SK-N-AS, and Kelly neuroblastoma cells were plated as above and treated with 5 μM 13-*cis*-retinoic acid, 1 μM of RXDX-105, or the combination of 5 μM 13-*cis*-retinoic acid and 1 μM of RXDX-105. Cells were treated for 72 hours and viability was assessed in each case using alamarBlue™ assays. Percent viability was obtained by normalizing each cell line and treatment to its respective vehicle control.

### Cell cycle analysis

Cell were treated for 24 hours with decreasing concentrations of RXDX-105 or equivalent concentrations of vehicle alone. Cells were then harvested and fixed in cold 70% ethanol and stored for at least 24 hours at −20° C. Prior to flow cytometry, cells were resuspended and rehydrated in PBS, treated with 100 μg/ml RNAse and then stained with 50 μg/ml propidium iodide. Samples were run on FACSCanto-II flow cytometer (BD Biosciences, Franklin Lakes, NJ, USA). Data was analyzed using FlowJo Software (TreeStar Inc., Ashland, OR, USA).

### Gene expression analysis

Using neuroblastoma cell line gene expression data [25] we performed oncoGPS analysis as previously described [35] on the Kelly, IMR-32, SK-N-AS, SK-N-BE (2), LA-N-5, SK-N-SH, CHP-134, and NGP neuroblastoma cell lines. Gene expression levels in the RAS signal transduction, BIOCARTA MAPK, positive regulation of MAPK Cascade, and KEGG MAPK signaling pathway gene sets from the Molecular Signatures Database [36] were evaluated in these 8 cell lines.

### Western blotting

Cells were plated in 6-well plates at approximately 70–80% confluence and allowed to adhere overnight. Wells were treated with RXDX-105 at different concentrations for 24 hours. RET phosphorylation was stimulated with 5 μM 13-*cis*-retinoic acid. Cells were then washed with PBS and lysed with RIPA buffer supplemented with Protease inhibitor and phosphatase inhibitor (Life Technologies, Carlsbad, CA, USA). Lysates were centrifuged and the supernatants were collected.

Xenograft tumors were snap frozen in liquid nitrogen at time of harvest (see below). Frozen tumor samples were broken up using a BioPulverizer (Biospec Products, Bartlesville, OK, USA) and lysed with RIPA buffer with 1% SDS, 10% protease inhibitor, and 10% phosphatase inhibitor.

Protein concentration in cell and tumor lysates was measured using a protein assay Dye Reagent (Bio-Rad, Hercules, CA, USA). 20–30 μg of denatured total protein from each sample was separated by sodium dodecyl sulfate-polyacrylamide gel electrophoresis (SDS-PAGE) using 4–12% Bolt gels (Invitrogen, Carlsbad, CA, USA) and transferred to PVDF membranes. Membranes were blocked with 3% BSA made in 1× TBS + 0.1% Tween 20 and then incubated with primary antibodies to total RET (#3220S), phospho-RET (#32212), total MEK1/2 (#9126S), phospho-MEK (#9154S), total ERK (#4695S), phospho-ERK (#4370S), PARP (#9542S), and GAPDH (#5174S) (all antibodies obtained from Cell Signaling Technology, Danvers, MA, USA). All antibodies were diluted in 5% BSA in 1× TBS + 0.01% Tween-20. Bound primary antibodies were incubated in anti-rabbit or anti-mouse HRP-conjugated secondary antibodies (1:5000, Sigma-Aldrich) at room temperature for 1 hour and the signal was visualized using Amersham ECL (GE Healthcare Bio-Sciences, Pittsburgh, PA, USA).

### Mouse tumor models

All mouse protocols were approved by the University of California, San Diego Institutional Animal Care and Use Committee (IACUC). Eight-week-old female athymic (*nu/nu*) mice were obtained from the UCSD Moores Cancer Center. Mice were anesthetized with the combination of ketamine and buprenorphine. 1 × 10^6^ luciferase-labelled SK-N-SH and SK-N-AS cells in 4 μL of PBS were injected into the exposed left adrenal glands of sedated mice. IVIS imaging was performed every 4 days, and once tumor signal was identified, mice were randomized and treated with either 80 mg/kg/day of RXDX-105 or equivalent volume of 22% HPBCD vehicle control by oral gavage. Animals were treated until tumors reached institutional limits, at which point all of the mice in the cohort were sacrificed and tumors were harvested. Tumor samples were divided, with half snap frozen in liquid nitrogen (see above) and half fixed in formalin (see below).

### Immunohistochemistry

Tumor samples were fixed in 1% Zn-Formalin for 24 hours then placed in 70% ethanol. Serial sections were used for immunohistochemistry. To assess vascular endothelial cells, frozen sections were incubated with rabbit anti-CD31 primary antibody (Abcam, Cambridge, UK; ab28364, 1:50) followed by anti-rabbit HRP-conjugated secondary antibody (Biocare, Pacheco, CA, USA; RMR 622). Sections were then stained with DAB (brown) chromogen (Biocare; IPK5010 G80) and Mayer’s hematoxylin (Sigma-Aldrich) counterstain and mounted with xylene based mountant. For calculation of mean vessel density, stained sections were assessed under light microscopy. Ten high-power field images (20×) were taken per section and the number of discrete vessels were counted per image in blinded fashion. Results are reported as average number of vessels per high power field.

### Statistical analysis

Experiments were performed at least in triplicate. Two tailed Student *t* tests were used to compare significance between means for *in vitro* and *in vivo* experiments.

## SUPPLEMENTARY MATERIALS



## Abbreviations

ERK: extracellular signal-related kinase
FBS: fetal bovine serum
GDNF: glial derived neurotrophic factor
MAPK: mitogen-activated protein kinase
MEK: MAPK/ERK kinase
PARP: poly ADP ribose polymerase
PBS: phosphate-buffered saline
PI: propidium iodide
RET: REarranged during Transfection
RTK: receptor tyrosine kinases
SDS: sodium dodecyl sulfate
SDS-PAGE: sodium dodecyl sulfate-polyacrylamide gel electrophoresis
VEGFR: vascular endothelial growth factor receptor
