# Supplementary material for: The multikinase inhibitor RXDX-105 is effective against neuroblastoma in vitro and in vivo
Source: Oncotarget. 2019 Oct 29;10(59):6323–33. doi: 10.18632/oncotarget.27259 (PMC6824878; doi:10.18632/oncotarget.27259)
Supplement: Supplementary file 1 [file oncotarget-10-6323-s001.pdf]

# The multikinase inhibitor RXDX-105 is effective against neuroblastoma *in vitro* and *in vivo*

## SUPPLEMENTARY MATERIALS

**Supplementary Table 1: Biological and cytogenetic features of neuroblastoma**

| Cell line | MYCN Status | 1p36 Del | 3p26 del | 11q23 del | 17q21-qter unbal gain | ALK mutation | P53 mutation |
|---|---|---|---|---|---|---|---|
| CHP-134 | Amplified | LOH p32.3-pter; Gain p34.3-p36.22; Loss p36.22-pter | Gain/AI p26.3 | None | Gain q12-qter | WT | WT |
| IMR-32 | Amplified | Loss p32.3-pter | Loss p12.3 | cnLOH q23.1 | Gain q21.2-qter | WT | WT |
| KELLY | Amplified | LOH p21.3-pter; Loss p36.32; Gain p36.33 | Loss p26.2 | Loss q23.3-qter | Gain q21.2-qter | F1174L | P177T |
| LA-N-5 | Amplified | Loss p33-pter | None | None | Gain q21.2-qter | R1275Q | WT |
| SJ-NB-10 | Amplified | Loss p32.2-pter | Gain p24.1-pter | cnLOH q23.1 | Gain q22-qter | WT; amplified | WT |
| NGP | Amplified | cnLOH p32.3-pter | Gain p25.3-pter | Loss q22.1-qter | Gain q21.1-qter | WT | A159D, C141W |
| SK-N-AS | Non-amplified | Loss p36.22-36.32 | Loss p14.2-pter | q13.4-qter | Gain q21.31-qter | WT | H168R |
| SK-N-BE(2) | Amplified | cnLOH p21.3-pter | Loss p14.2-pter | Gain/AI q13.1-qter | Gain q12-qter | WT | C135F |
| SK-N-SH | Non-amplified | None | None | None | Gain q21.31-qter | F1174L | WT |

Adapted from Harenza *et. al*. [25].

**Supplementary Table 2: IC50 values for inhibition of cell proliferation**

| Cell line | IC 50 [µM] |
|---|---|
| Kelly | 3.5 |
| IMR-32 | 4.3 |
| SK-N-AS | 5.6 |
| SK-N-BE(2) | 7.8 |
| LA-N-5 | 9.9 |
| SK-N-SH | 10.4 |
| LA-155-N | 12.1 |
| LA-N-1 | 12.4 |
| CHP-134 | 15.1 |
| SJ-NB-10 | 15.4 |
| NGP | 15.9 |

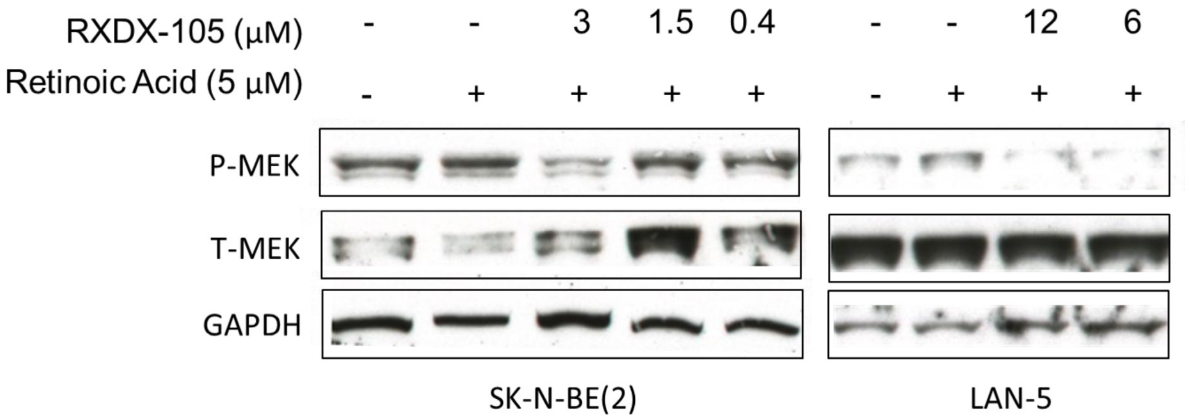


**Supplementary Figure 1: RXDX-105 inhibits target kinases and intracellular signaling pathways.** Neuroblastoma cell lines were treated with either vehicle or decreasing doses of RXDX-105 for 24 hours in combination with 5 µM 13-*cis*-retinoic acid. Cells were lysed and Western blots were performed to assess MEK expression and phosphorylation. GAPDH expression is shown as a loading control.

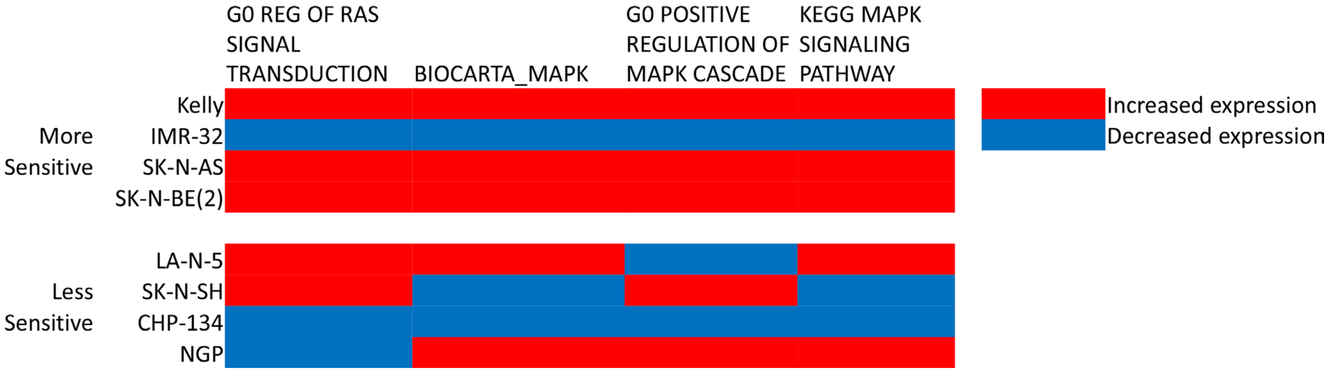


**Supplementary Figure 2: Sensitivity to RXDX-105 is related to RAS-MAPK gene expression.** Neuroblastoma cell line gene expression from [25] was assessed to evaluate for differences in gene pathway expression using pathways from the Molecular Signatures Database [36] underlying relative cell line sensitivities to RXDX-105. Cell lines with IC50 values less than 8 µM were considered more sensitive, while cell lines with IC50 values above 8 µM were considered less sensitive. Increased pathway gene expression is shown in red, while decreased pathway gene expression is shown in blue.

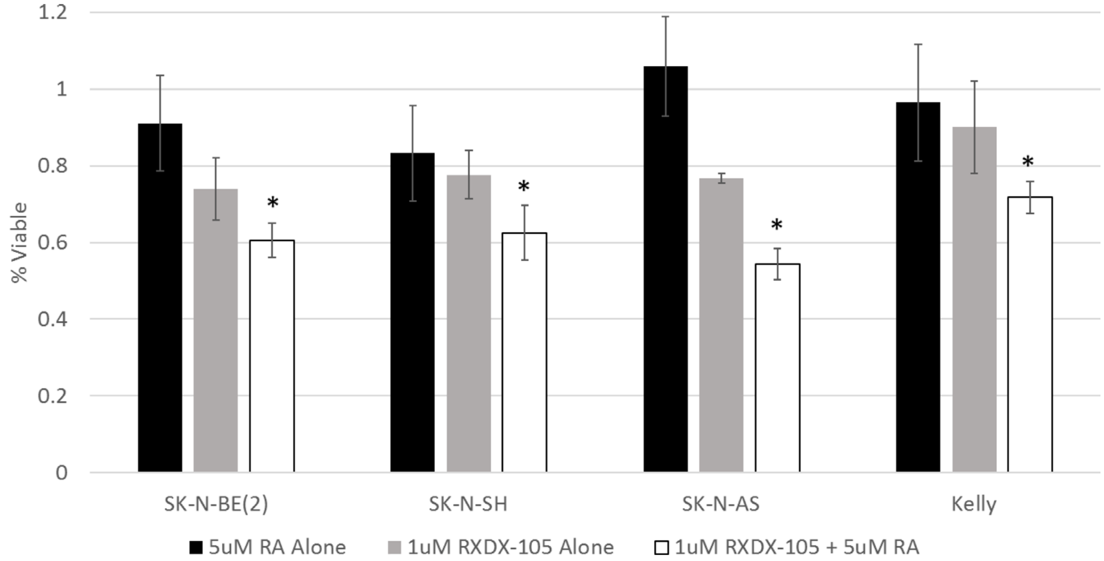


**Supplementary Figure 3: RXDX-105 efficacy is enhanced when combined with 13-*cis*-retinoic acid.** A panel of neuroblastoma cell lines were treated with 1 µM RXDX-105 alone or in combination with 5 µM 13-*cis*-retinoic acid for 72 hours and cell viability was assessed with an alamarBlue™ assay. Cell viability with each condition is shown.

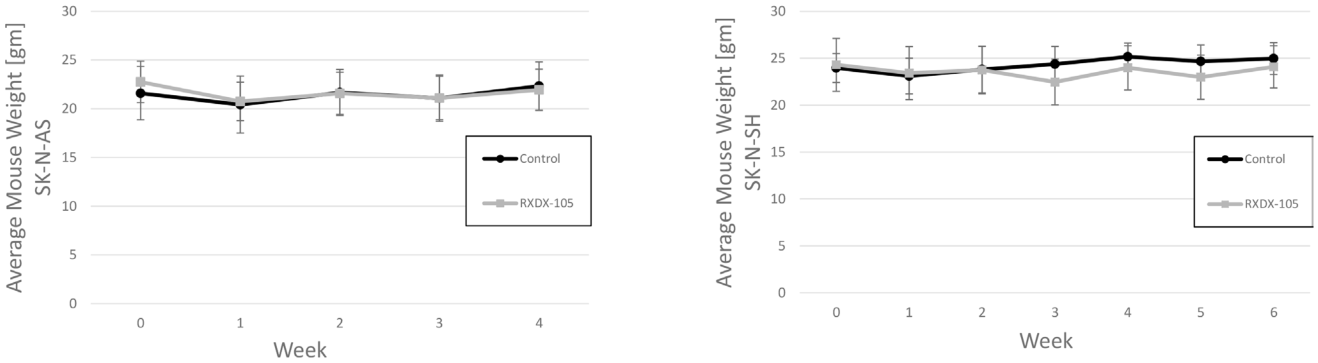


**Supplementary Figure 4: Mouse weight during RXDX-105 treatment.** Mice being treated with RXDX-105 or vehicle control were weighed weekly during treatment and weights were plotted on graphs.

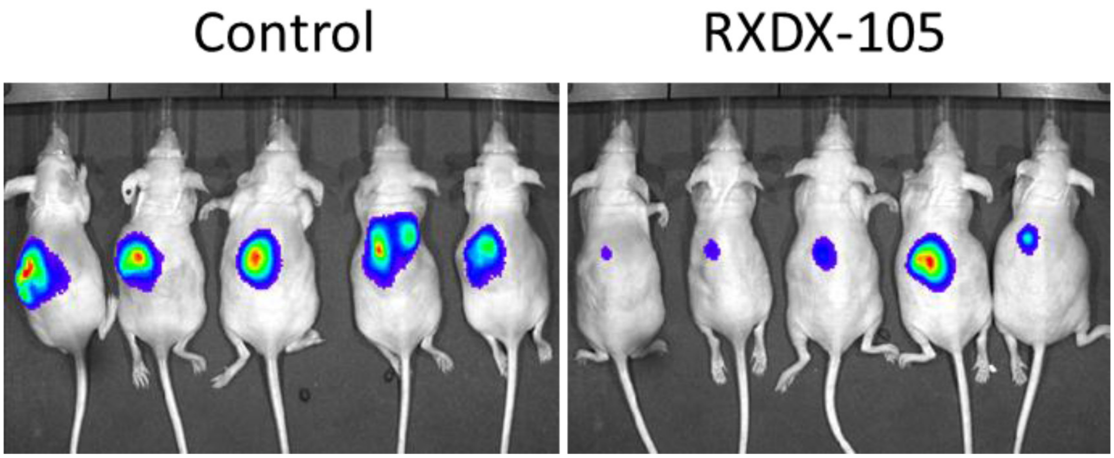


**Supplemental Figure 5: RXDX-105 inhibits tumor xenograft growth.** Bioluminescent images of orthotopic neuroblastoma xenograft tumors in mice treated for six weeks with vehicle alone (left) or with RXDX-105 (right).
